# Evaluation of the Cross-Protective Efficacy of a Chimeric Porcine Reproductive and Respiratory Syndrome Virus Constructed Based on Two Field Strains

**DOI:** 10.3390/v8080240

**Published:** 2016-08-22

**Authors:** Nadeem Shabir, Amina Khatun, Salik Nazki, Bumseok Kim, Eun-Jin Choi, Dong Sun, Kyoung-Jin Yoon, Won-Il Kim

**Affiliations:** 1 College of Veterinary Medicine, Chonbuk National University, Iksan 54596, Korea; drnadurose@gmail.com (N.S.); amina.vet.sau.bd@gmail.com (A.K.); saliknazki@jbnu.ac.kr (S.N.); bskims@jbnu.ac.kr (B.K.); 2 Viral Disease Division, Animal and Plant Quarantine Agency, Anyang 430-757, Korea; choiej@korea.kr; 3 Department of Veterinary Diagnostic and Production Animal Medicine, College of Veterinary Medicine, Iowa State University, Ames, IA 50011, USA; dong.sun@zoetis.com (D.S.); kyoon@iastate.edu (K.-J.Y.)

**Keywords:** porcine reproductive and respiratory syndrome, chimeric virus, cytokines, serum virus neutralizing antibodies, vaccine, pig

## Abstract

One of the major hurdles to porcine reproductive and respiratory syndrome (PRRS) vaccinology is the limited or no cross-protection conferred by current vaccines. To overcome this challenge, a PRRS chimeric virus (CV) was constructed using an FL12-based cDNA infectious clone in which open reading frames (ORFs) 3–4 and ORFs 5–6 were replaced with the two Korean field isolates K08-1054 and K07-2273,respectively. This virus was evaluated as a vaccine candidate to provide simultaneous protection against two genetically distinct PRRS virus (PRRSV) strains. Thirty PRRS-negative three-week-old pigs were divided into five groups and vaccinated with CV, K08-1054, K07-2273, VR-2332, or a mock inoculum. At 25 days post-vaccination (dpv), the pigs in each group were divided further into two groups and challenged with either K08-1054 or K07-2273. All of the pigs were observed until 42 dpv and were euthanized for pathological evaluation. Overall, the CV-vaccinated group exhibited higher levels of tumor necrosis factor-alpha (TNF-α), interferon-gamma (IFN-γ), and interleukin-12 (IL-12) expression and of serum virus-neutralizing antibodies compared with the other groups after vaccination and also demonstrated better protection levels against both viruses compared with the challenge control group. Based on these results, it was concluded that CV might be an effective vaccine model that can confer a broader range of cross-protection to various PRRSV strains.

## 1. Introduction

More than two decades after its emergence in the United States and Europe, porcine reproductive and respiratory syndrome (PRRS) continues to be a cause of great concern for swine producers worldwide. The disease is characterized by stillbirth and abortion in adult pigs and respiratory disease and mortality in pigs of all ages [1]. The causative agent, porcine reproductive and respiratory syndrome virus (PRRSV), is a single-stranded positive sense RNA virus (~15 kb) that belongs to the family *Arteriviridae*andorder *Nidovirales* [2]. PRRSV has a polycistronic genome that consists of two large open reading frames (ORFs), 1a and 1b, encoding non-structural proteins (nsps) and eight ORFs that encode structural proteins [3,4]. ORFs 1a and 1b encode replicase polyproteins 1a (pp1a) and pp1ab, and translation of the latter occurs due to a ribosomal frame shift towards the C-terminus of ORF1a. These polyproteins are serially cleaved into at least 14 nsps [5]. ORFs 2a, 3, and 4 encode three *N*-glycosylated minor envelope proteins, viz. GP2a, GP3, and GP4, which are linked by disulfide bonds to form heterotrimers [6]. ORF2b, embedded in ORF2a, encodes a non-glycosylated minor protein [7]. GP5, encoded by ORF5, is a major envelope protein that forms a heterodimer with matrix (M) protein, which is encoded by ORF6 [8]. This heterodimer recruits other envelope protein heterodimers and thus plays an important role in virus assembly [6]. Additionally, GP5a has been recently identified and is encoded by an alternate ORF5 [4]. The nucleocapsid (N) protein is expressed from ORF7 and contains nuclear localization signals (NLSs) and nuclear export signals (NESs) [9].

Presently, a major drawback in the use of current PRRS modified live vaccines (MLVs) is that they offer limited to no cross-protection despite conferring solid homologous protection [10,11,12,13]. Killed PRRS vaccines have also been previously used, but they offer limited protection against even homologous viruses [3]. It is now generally accepted that strain-specific vaccines cannot fully protect against newly emerging PRRSV outbreaks, whereas multi-strain vaccines have shown more severe clinical signs in pigs [14]. Moreover, owing to the considerable variation between PRRSV strains in terms of replication efficiency [15], immune response [16], and neutralizing antibody induction [17], multi-strain vaccines for PRRS have limited potential. Conventional methods used for RNA virus vaccine development have been successful in the past, but reverse genetics approach might be more efficient [18]. To date, over 14 infectious clones of type I and type II PRRSV have been developed using reverse genetics, and this approach can be further utilized to construct safe and efficacious chimeric vaccines against PRRSV [5,19]. Recently, a study revealed that vaccine chimeras between MN-184 and licensed MLV (Ingelvac^®^ PRRS MLV) attenuated clinical signs in pigs challenged with virulent viruses [20]. However, to broaden the cross-protective range of vaccine candidates, the chimerization of multiple PRRSV strains should be attempted to develop a monovalent chimeric vaccine that includes neutralizing epitopes from genetically diverse PRRSV strains. Therefore, in this study, a chimeric virus (CV) derived from three genetically diverse strains of PRRSV was constructed and evaluated for its safety and cross-protective efficacy.

## 2. Materials and Methods

### 2.1. Viruses and Cells

VR-2332, a parental virus of MLV (Ingelvac^®^ PRRS MLV, Boehringer Ingelheim, St. Joseph, MO, USA), which is the most commonly used vaccine in Korea, and two type 2 Korean PRRSV strains, K08-1054 (Accession number: JQ656266) and K07-2273 (Accession number: JQ656251), were used in this study. Based on the ORF5 amino acid sequence, K08-1054 and K07-2273 share 85.1% identity, and FL12 and K08-1054 or K07-2273 share 91% or 88.1% identity, respectively. VR-2332 shares a high sequence identity (96.5%) with K08-1054 and a low identity (84.6%) with K07-2273, whereas FL12 and VR-2332 share 90.5% sequence identity (Table 1). MARC-145, an African green monkey kidney cell line that is highly permissive to PRRSV, was used for virus propagation. MARC-145 cells were maintained in an Roswell Park Memorial Institute (RPMI)-1640 medium (Gibco^®^ RPMI 1640, Life Technologies, Carlsbad, CA, USA) supplemented with heat-inactivated 10% fetal bovine serum (FBS, Life Technologies), 2 mM l-glutamine, and Antibiotic-Antimycotic 100× (Anti-anti, Life Technologies) containing 100 IU/mL penicillin, 100 µg/mL streptomycin, and 0.25 µg/mL Fungizone^®^ (amphotericin B). In this paper, this medium will be referred to as complete RPMI (cRPMI) medium. The cells were maintained at 37 °C in a humidified 5% CO_2_ incubator.

### 2.2. Construction of Chimeric Infectious Clones

**Figure 1:**
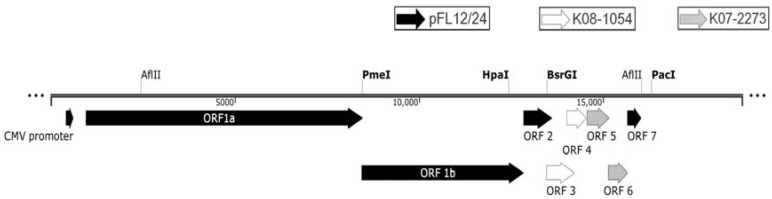
Graphical representation of the genomic construct for the chimeric infectious clone (pFLK_3–6_). Black, white, and gray arrows represent the open reading frames (ORFs)/gene fragments from pFL12/24, K08-1054, and K07-2273, respectively. The restriction sites used for cloning are listed above the construct. CMV: human cytomegalovirus; AflII, BsrGI, HpaI, PacI, PmeI: restriction sites.

FL12 cDNA infectious clone [21] was re-cloned into a vector (pOptiVEC™-TOPO^®^ TA Cloning kit, Life Technologies) to construct a modified FL12-based infectious clone (pFL12/24) by inserting FL12 viral sequences between the human cytomegalovirus (CMV) promoter and the internal ribosomal entry site (IRES) that are present in the vector. Because the modified infectious clone contains the human CMV promoter and an IRES that initiates transcription and translation of the viral genome, viruses can be rescued after direct transfection of the infectious clone into MARC-145 cells without in vitro transcription [19]. Then, the structural genes (ORFs 3–7) including the downstream end of pFL12/24 ORF2 were cloned into a vector (pGEM^®^-T Easy vector, Promega Corporations, Madison, WI, USA) using SphI and SpeI restriction sites to produce an FL12-based structural gene shuttle vector (sFL_3–7_). The shuttle vector was used as a template for gene swapping using PCR-based mutagenesis as described in a previous study [22]. ORFs 3–4 and 5–6 of sFL_3–7_ were sequentially replaced with the corresponding ORFs from K08-1054 and K07-2273, respectively, to construct a chimeric shuttle vector (sFLK_3–6_). Then, ORFs 1b–3 from pFL12/24 were digested and cloned into the chimeric shuttle vector using PmeI and BsrGI restriction sites to construct sFL1b-K_3–6_^+^. Because a major portion of ORF3 was duplicated in this chimeric shuttle vector, the 1047 bp region between the HpaI and BsrGI restriction sites was amplified from pFL12/24 using high-fidelity PCR (GeneAmp^®^ High Fidelity PCR system, Applied Biosystems^®^, Life Technologies) to replace the ORF three-repeat region (1813 bps) in sFL1b-K_3–6_^+^ using HpaI and BsrGI restriction sites*,* which consequently led to the construction of sFL1b-K_3–6_. Then, the sequences downstream from ORF 6, including the IRES sequence downstream of the end of the pFL12/24 poly-A tail, were amplified with high-fidelity PCR (GeneAmp^®^ High Fidelity PCR system) using the primers listed in Table 1 and were cloned into sFL1b-K_3–6_ using AflII and PacI restriction sites, generating sFL1b-K_3–6_R. ORF1b, the structural genes and the IRES sequence from sFL1b-K_3–6_R, were placed in the pFL12/24 backbone to construct the chimeric infectious clone pFLK_3–6_ (Figure 1). All of the primers used to construct pFLK_3–6_ are shown in Table 2.

### 2.3. Generation of Mutant Virus

After confirming the sequence of ORF1b and all of the structural genes, the chimeric infectious clone pFLK_3–6_ was transfected into MARC-145 cells. Cells were harvested, washed, and resuspended in Dulbecco’s modified Eagle medium (DMEM) containing 1.25% dimethyl sulfoxide (DMSO) at 5 × 10^6^ cells/mL. The cells were transfected with 10µg of chimeric infectious clone and were electroporated at 250 V and 950 μF using an electroporation system (Gene PulserXcell™ Electroporation System, Bio-Rad Laboratories, Hercules, CA, USA). After electroporation, the cells were diluted in cell growth medium without antibiotics, seeded into six-well plates (BD Falcon, Franklin Lakes, NJ, USA) and incubated in a humidified chamber at 37 °C and 5% CO_2_. After 16 h of incubation, the cells were replenished with cRPMI and incubated under the same conditions for 32 h, after which the supernatant was collected. The MARC-145 cells seeded in 24-well plates were infected with 0.5 mL of the supernatant and were incubated under same conditions for up to five days until cytopathic effect (CPE) was observed in the cells. CV stocks were prepared by passaging supernatants three times in MARC-145 cells and were stored at −80 °C after titration until use. The rescued virus was sequenced, and the full-length CV sequence was deposited in GenBank (Accession number: KP70428).

### 2.4. Design for Animal Experiments and Sample Collection

A total of 30 three-week-old PRRS-negative pigs were purchased and randomly assigned to five groups of six each. Group 1 was mock-treated with RPMI, and groups 2–5 were vaccinated intramuscularly (IM) with K07-2273, K08-1054, VR-2332, or CV, respectively, using 2 mL of each virus diluted in RPMI to 10^3^ 50% tissue culture infectious dose (TCID_50_)/mL. At 25 days post-vaccination (dpv), each group of six pigs was randomly divided into two groups of three and challenged with either K08-1054 or K07-2273 at a titer of 10^3^ TCID_50_/mL by the intranasal (IN) route. Serum samples from all of the pigs were collected every week until 21 dpv, after which they were collected every three days after virus challenge until 42 dpv or 17 days post-challenge (dpc). Whole blood was collected at 21 dpv for peripheral blood mononuclear cell (PBMC) isolation. All of the pigs were weighed at 0 and 25 dpv and euthanized for necropsy at 42 dpv. To evaluate gross and microscopic lung lesions, each lung lobe was scored for percentage of lung consolidation [23] and interstitial pneumonia, respectively, caused by PRRSV infection. Scoring for microscopic lung lesions was recorded as follows: 0, indicates no lesion; 1, mild interstitial pneumonia; 2, moderate multifocal interstitial pneumonia; 3, moderate diffused interstitial pneumonia; and 4, severe interstitial pneumonia. Lung tissues were collected from each pig and stored at −80 °C until examination. The animal experimental protocol was approved by the Chonbuk National University Institutional Animal Care and Use Committee (Approval number: 2012-0025).

### 2.5. Quantification of PRRSV RNA in Sera and Lungs

Viral RNA was extracted from 100 µL of serum and 1g of lung samples using a viral RNA extraction kit (MagMAX™ Viral RNA Isolation Kit, Life Technologies) and a total RNA extraction kit (Hybrid-RTM, GeneAll, Seoul, Korea) according to the manufacturer’s instructions. The virus levels in serum and lungs were measured using a real-time reverse transcription-polymerase chain reaction (RT-PCR) employing a one-step reverse transcriptase kit (AgPath-IDTM One-Step RT-PCR Kit, Ambion, Austin, TX, USA) with the 7500 Fast Real-time PCR system (Applied Biosystems, Foster City, CA, USA). Primers and aminor groove binder (MGB) fluorescent probe specific to a conserved region of ORF7 were used as described previously [24].

### 2.6. Assessment of PRRSV-Specific Antibodies

A fluorescent focus neutralization (FFN)-based serum virus neutralization (SVN) assay was performed to evaluate SVN antibody titers induced by PRRSVs after vaccination and challenge. The cross-SVN antibodies induced in vaccinated and challenged groups were evaluated against K08-1054 and K07-2273. The SVN assay was performed in MARC-145 cells as described previously [7,22]. The SVN antibody titer of each anti-serum against each virus was expressed as the reciprocal of the highest dilution in which 90% or more reduction in the number of fluorescent focus unit (FFU) was observed compared to the wells of the respective virus back titration.

The presence of PRRSV N-specific antibodies in the sera of vaccinated and virus-challenged animals was determined using a direct enzyme-linked immune sorbent assay (ELISA) kit (HerdCheck^®^PRRS Antibody Kit 3XR, IDEXX Laboratories, Westbrook, ME, USA) according to the manufacturer’s instructions.

### 2.7. Isolation of PBMCs

Blood from each pig was collected 21 days after vaccination, and the isolation of porcine peripheral blood mononuclear cells was performed using the density gradient method in Histopaque-1077^®^ solution (Sigma, St. Louis, MO, USA) from 5mL blood samples collected in lithium-heparin-containing vacutainers according to the manufacturer’s instructions. The blood samples were briefly stratified on Histopaque-1077^®^ solution at a ratio of 1:1 (blood: Histopaque) and centrifuged at 400× *g* for 30 min. The purified PBMCs were collected and washed twice with sterile phosphate buffered saline (PBS) (pH 7.0) supplemented with 1% FBS (Gibco, Carlsbad, CA, USA) and were resuspended in 0.5 mL of sterile PBS. To evaluate viability and number, the cells were diluted 100 times in PBS, mixed with 0.4% Trypan blue at a 1:1 ratio, and counted using a Countess™ Automated Cell Counter (Invitrogen, Carlsbad, CA, USA). The cells were diluted to 1 × 10^6^ cells/mL in cRPMI, and 1 mL of cells per well were seeded in 24-well plates (BD Falcon, Franklin Lakes, NJ, USA) and incubated at 37 °C in a humidified 5% CO_2_ incubator for 72 h.

### 2.8. Cytokine mRNA Quantification by Real-time RT-PCR

PBMCs were harvested at 72 h, and cellular RNA was extracted using an RNA isolation kit (GeneAll^®^ Hybrid-RTM kit, GeneAll Biotechnology, Seoul, Korea) following the manufacturer’s instructions. RNA was reverse-transcribed into complementary DNA (cDNA) using a high-capacity cDNA reverse transcription kit (Applied Biosystems) following the manufacturer’s instructions. Real-time RT-PCR was performed on a 7500 Fast Real-time PCR system (Applied Biosystems) using various cytokine-specific primers according to the manufacturer’s instructions. Sequences of the primers used in this study are shown in Table 3. Ten microliters of 2× Power SYBGR (Applied Biosystems), 2 μL cDNA, and 1 μL each of the forward primer (10 pmol/μL) and reverse primer (10 pmol/μL) were used for PCR amplification. All of the samples were tested in duplicate, and the cycling conditions were as follows: (a) 10 min at 95 °C; (b) 40 cycles of 15 s at 95 °C and 1 min at 60 °C; and (c) melt curve stage for 15 s at 95 °C, 1 min at 60 °C, 15 s at 95 °C, and 15 s at 60 °C. Relative quantities of cytokine mRNA in infected and non-infected cells were normalized to β-actin mRNA, and the amounts were determined using the 2^−ΔΔCt^ method [25].

### 2.9. Cytokine Quantification by ELISA

Tumor necrosis factor-α (TNF-α) and interleukin-12 (IL-12) protein levels in sera and PBMC supernatants were quantified using a commercially available ELISA kit (DuoSet^®^ ELISA, R&D Systems, Minneapolis, MN, USA) according to the manufacturer’s instructions.

### 2.10. Sequence and Data Analysis

Graphs were constructed using GraphPad Prism 5.0.2 (GraphPad, San Diego, CA, USA), and statistical analysis was performed using SPSS Advanced Statistics 17.0 software (SPSS Inc., Chicago, IL, USA). The Mann-Whitney *U* test was used to estimate the differences between groups. To prepare a consensus sequence, the forward and reverse nucleotide sequences were aligned using Seqman™ (DNASTAR Inc., Madison, WI, USA). The consensus sequences of different viruses were aligned using Lasergene^®^ MegAlign software (DNASTAR Inc.). The phylogenetic tree was generated using a neighbor-joining method available in the Molecular Evolutionary Genetic Analysis software package, version 6 (MEGA6) [27].

## 3. Results

### 3.1. Safety of Chimeric Vaccine

**Figure 2:**
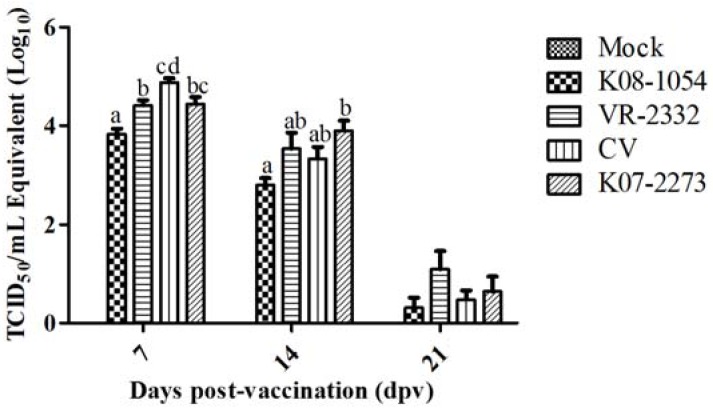
Viral loads in pig serum at 0, 7, 14, and 21 days post-vaccination (dpv). The virus titers were calculated based on the standard curve of the threshold cycle number plotted against the known virus titer of VR-2332. The bars represent the means, and the error bars represent the standard errors of the mean (SEM). Bars showing different letters represent values significantly different from each other (*p* < 0.05). TCID_50_: 50% tissue culture infectious dose. CV: chimeric virus.

**Figure 3:**
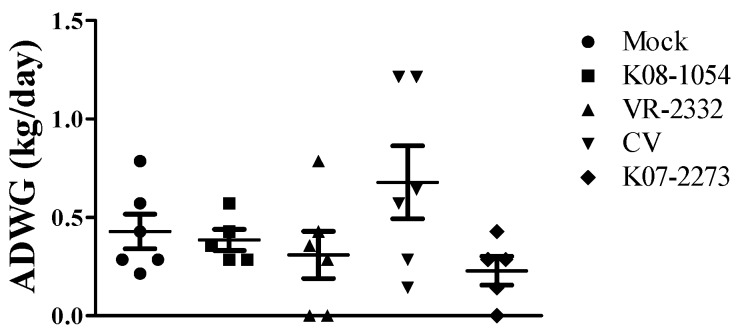
Effect of virus replication on pig growth. The average daily weight gain (ADWG) was calculated from six pigs in each group at 25 dpv. The bars represent the means, and the error bars represent the standard errors of the mean (SEM).

The mean peak virus titers in all of the vaccinated groups ranged between 10^3.82^ and 10^4.87^ TCID_50_/mL at 7 dpv, which gradually declined to 10^0.3–1.0^ TCID_50_/mL at 21 dpv (Figure 2). The CV showed significantly higher replication in pigs at 7 dpv compared with the other vaccination groups, reaching a mean peak titer of 10^4.87^ TCID_50_/mL and decreasing to 10^0.47^ TCID_50_/mL at 21 dpv. The other viruses (K08-1054, VR-2332 and K07-2273) reached mean peak titers of 10^3.82^, 10^4.41^ and 10^4.43^ TCID_50_/mL, respectively, at 7 dpv and gradually declined to 10^0.31^, 10^1.09^ and 10^0.64^ TCID_50_/mL at 21 dpv. However, the average daily weight gain (ADWG) was highest in the CV-vaccinated group when gains were assessed between 0 and 25 dpv to evaluate the effect of the vaccines on pig growth, although the difference was not significant (Figure 3).

### 3.2. Cytokine Responses by Chimeric Vaccine Inoculation

**Figure 4:**
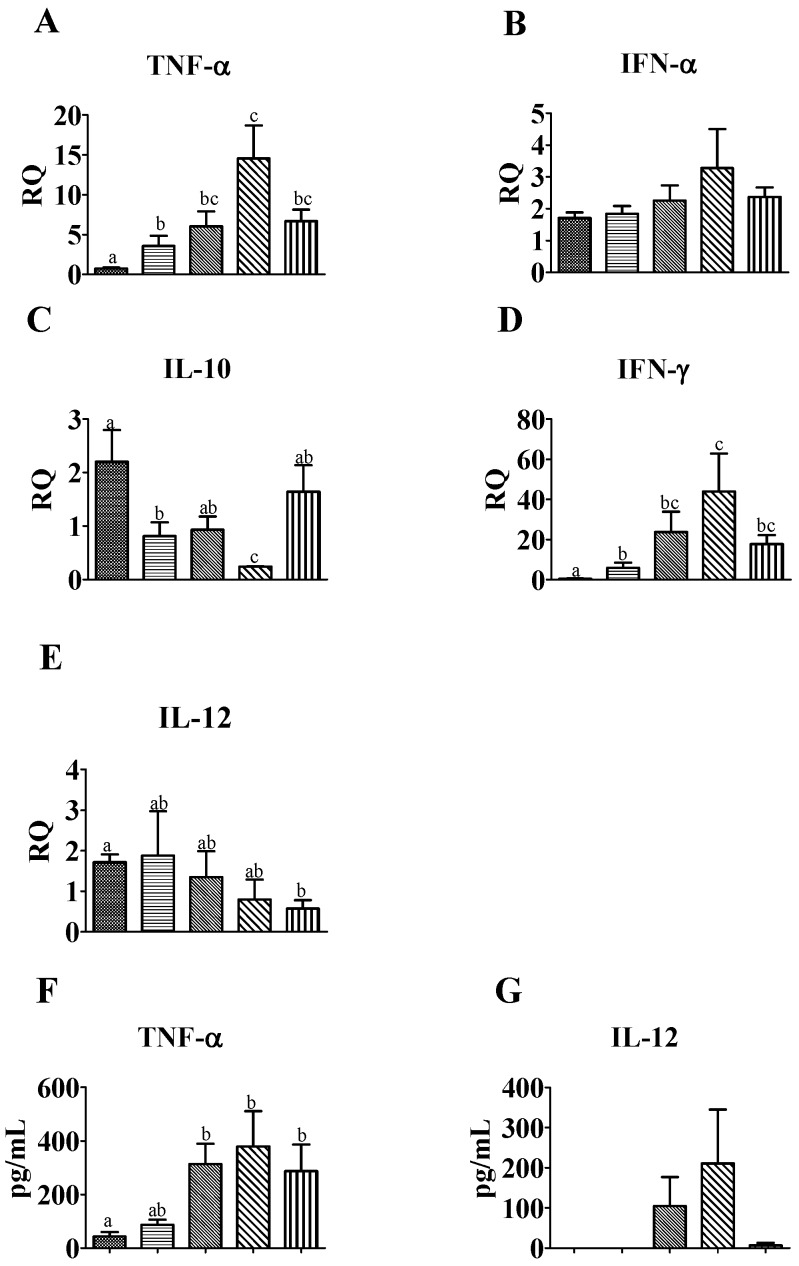
mRNA and protein expression of cytokines in peripheral blood mononuclear cells (PBMCs) from vaccinated pigs at 21 dpv. Analysis of interferon-alpha (IFN-α), IFN-γ, interleukin (IL)-10, IL-12, and tumor necrosis factor-alpha (TNF-α) mRNAs (**A**–**E**) and protein (**F**,**G**) expression levels of various cytokines in PBMCs after 72 h culture. Changes in mRNA expression were evaluated by quantitative real time polymerase chain reaction (qRT-PCR). Relative quantification of mRNA expression was estimated using the 2^−ΔΔCt^ method, and IL-12 and TNF-αprotein levels were quantified in PBMC supernatants by ELISA. Bars showing different letters represent values significantly different from each other (*p* < 0.05). RQ: Relative Quantification.

The mRNA and protein expression levels of various cytokines were quantified in the PBMCs of vaccinated pigs (Figure 4). mRNA encoding the regulatory cytokine IL-10 was significantly lower in the CV-vaccinated group compared to any other group. In contrast, interferon-γ (IFN-γ) and TNF-α mRNA levels were highest in the CV-vaccinated group among all of the groups and were significantly higher (*p* < 0.05) than those of mock-treated control and K08-1054-vaccinated groups. TNF-α protein levels in the supernatant of PBMC cultures from the CV group were the highest of the groups and were significantly higher than the mock-treated control group (*p* < 0.05). There were no significant differences in IFN-α and IL-12 mRNA levels among the groups, and IL-12 protein levels in the PBMC supernatant were detected in only two out of six pigs each in the VR-2332- and CV-vaccinated groups.

### 3.3. Antibody Responses after Vaccination and Challenge

**Figure 5:**
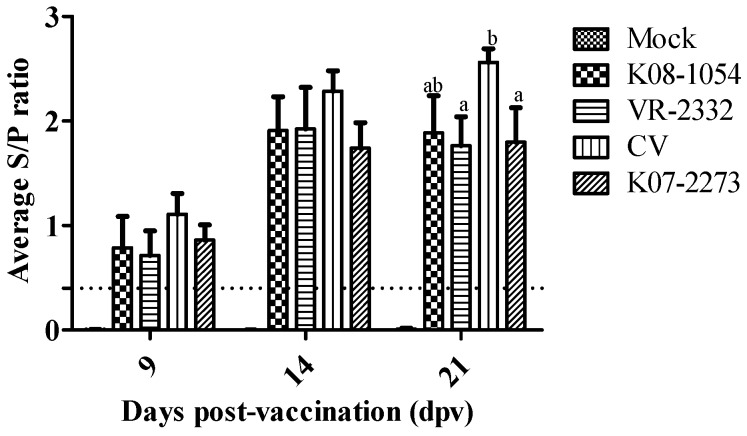
Antibody titers of vaccinated pigs measured by PRRS ELISA. The 0.4 S/P ratio represents the threshold value designated by a dashed line. Bars showing different letters represent values significantly different from each other (*p* < 0.05).

Animals in all of the groups except the non-vaccinated group tested positive by IDEXX PRRS ELISA at 7 dpv (Figure 5). The sample-to-positive (S/P) values in CV-vaccinated pigs were higher than any other group at 7 dpv and 14 dpv and were significantly higher (*p* < 0.05) than the S/P values in VR-2332- and K07-2273-vaccinated pigs at 21 dpv.

**Figure 6:**
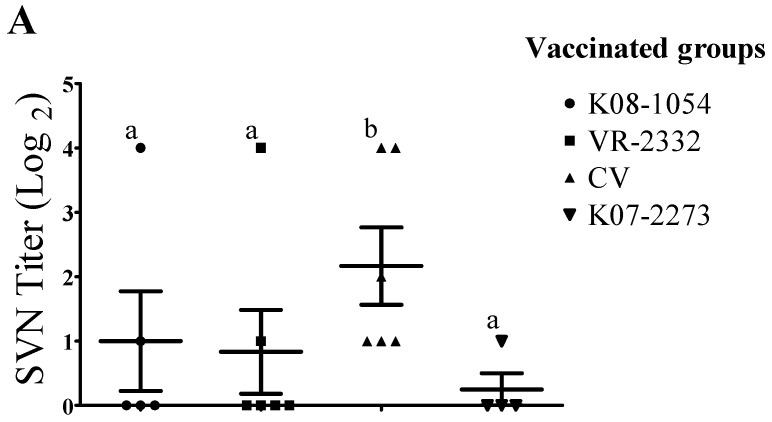

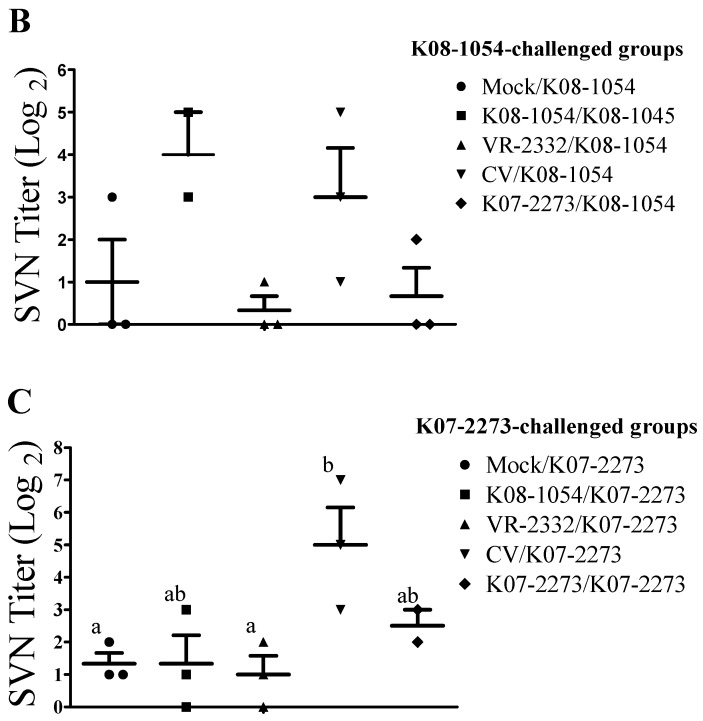
Serum virus neutralization (SVN) antibody titers of vaccinated pigs 21 and 42 dpv. SVN antibody titers of sera collected from pigs vaccinated with K08-1054, VR-2332, CV or K07-2273 against each homologous virus at 21 dpv (**A**). Virus-neutralizing antibody titer in sera from K08-1054-challenged groups against K08-1054 at 42 dpv (**B**) and virus-neutralizing antibody titer in sera from K07-2273-challenged groups against K07-2273 at 42 dpv (**C**). Bars showing different letters represent values significantly different from each other (*p* < 0.05).

Based on the virus neutralization test results, pigs inoculated with CV produced approximately 2- to 5-fold higher (*p* ≤ 0.05) SVN antibody titers compared with pigs inoculated with K08-1054, VR-2332, or K07-2273 against each homologous virus at 21 dpv (Figure 6A). However, no pigs from either of the inoculated groups produced SVN antibody against heterologous viruses at 21 dpv (data not shown). The SVN antibody titers in all of the groups were evaluated again 42 dpv after the virus challenge. The SVN antibody titers induced in CV/K08-1054 were very similar to the SVN antibody titers induced in the homologous challenge group (K08-1054/K08-1054) (*p* > 0.05) and approximately 3- to 6-fold higher than Mock/K08-1054, VR-2332/K08-1054, or K07-2273/K08-1054 against K08-1054 (Figure 6B). Moreover, among the groups challenged with K07-2273, CV/K07-2273 produced the highest SVN antibody titres, which were approximately 3- to 5-fold higher than those of Mock/K07-2273, K08-1054/K07-2273 or VR-2332/K07-2273 and were also 3-fold higher than those of the homologous challenge group (K07-2273/K07-2273) against K07-2273 (Figure 6C).

### 3.4. Viremia Levels after Challenge

**Figure 7:**
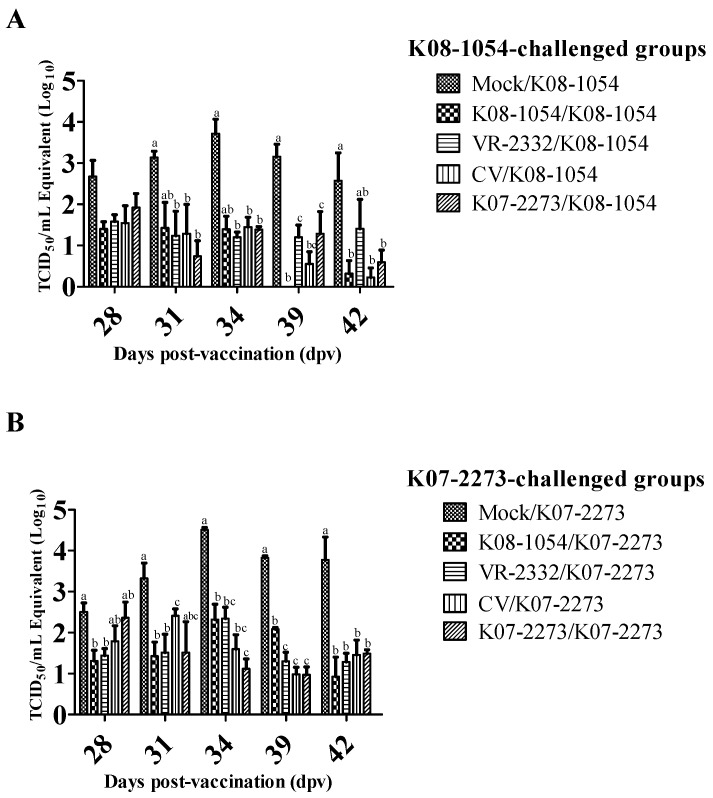
The levels of viremia after virus challenge at 28, 31, 39 and 42 dpv. Serum virus titers of K08-1054-challenged groups at 28, 31, 34, 39, and 42 dpv (**A**) and serum virus titers of K07-2273-challenged groups at 28, 31, 34, 39, and 42 dpv (**B**). The virus titers were calculated based on the standard curve of threshold cycle number plotted against the known virus titer of VR-2332. The bars represent the means, and the error bars represent the standard errors of the mean (SEM). Bars showing different letters represent values significantly different from each other (*p* < 0.05).

After non-vaccinated pigs were challenged with K08-1054, acute-phase viremia was observed at 34 dpv with a peak virus titer of 10^3.71^ that gradually decreased to 10^2.54^ TCID_50_/mL by 42 dpv. The homologous challenge (K08-1054/K08-1054) and chimeric virus (CV/K08-1054) groups developed mild viremia after the challenge that steadily decreased and was nearly resolved at 42 dpv. The other two heterologous groups (K08-1054/K07-2273 and K08-1054/VR-2332) also developed mild viremia. However, K08-1054/K07-2273 experienced delayed viremia reduction, whereas there was negligible virus titer decrease in the K08-1054/VR-2332 group from 28 dpv to 42 dpv (Figure 7A).

Non-vaccinated pigs challenged with K07-2273 also developed acute-phase viremia that reached a peak virus titer of 10^4.50^ TCID_50_/mL at 34 dpv but declined steadily to 10^3.77^ TCID_50_/mL by 42 dpv. The homologous challenge (K07-2273/K07-2273) and the chimeric virus (CV/K07-2273) groups challenged with K07-2273 displayed a similar pattern of viremia, although there was a slight increase in the virus titer in the CV/K07-2273 group at 31 dpv (Figure 7B). The average virus titer for the VR-2332/K07-2273 group steadily increased to 10^2.34^ TCID_50_/mL at 34 dpv and decreased to a level similar as the homologous challenge group by 39 and 42 dpv. However, the average virus titer for the K08-1054/K07-2273 group increased to 10^2.2^ TCID_50_/mL at 31 dpv, was constant until 39 dpv, and decreased to 10^0.92^ TCID_50_/mL at 42 dpv.

### 3.5. Pathology and Viral Load in Lungs

**Figure 8:**
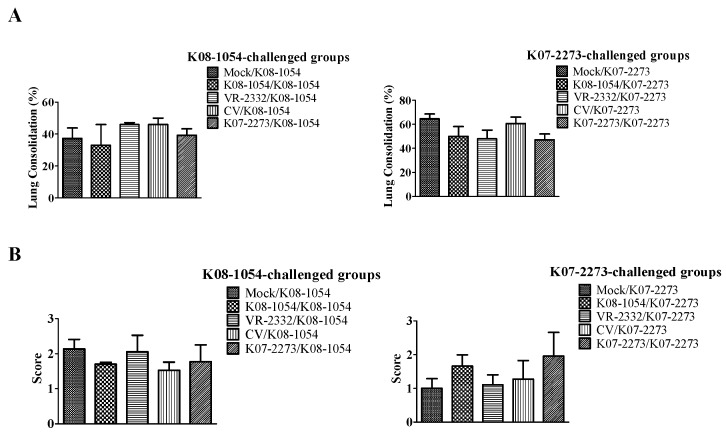
Evaluation of gross and microscopic lung lesions in pigs at 42 dpv. Gross (**A**) and microscopic (**B**) lung scores were recorded after necropsy. Lung scores were plotted as the mean lesion score values from three pigs in each group, and the error bars represent the standard error of the mean (SEM).

**Figure 9:**
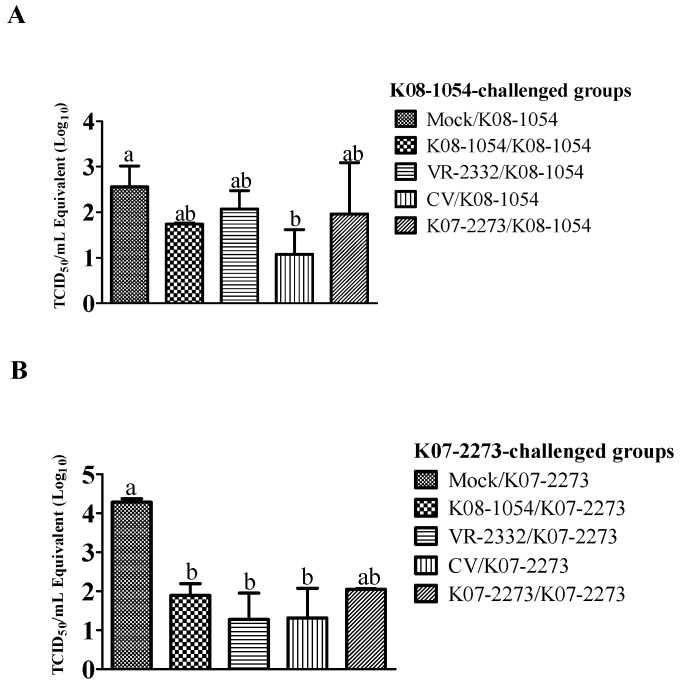
Quantification of residual viral lung load in challenged pigs at 42 dpv. Residual viral load in the lungs of K08-1054-challenged groups at 42 dpv (**A**); Residual viral load in the lungs of K07-2273-challenged groups at 42 dpv (**B**). The virus titers were calculated based on the standard curve of the cycle threshold number plotted against the known virus titer of VR-2332. The bars represent the means, and the error bars represent the standard error of the mean (SEM). Bars with different letters depict values significantly different from each other (*p* < 0.05).

There were no significant differences in gross or microscopic lung lesion scores among the groups challenged with K08-1054 or K07-2273 (Figure 8). To obtain better insight regarding viral clearance from the pigs’ bodies, the residual viral load in the lungs was determined after euthanizing all of the pigs at 42 dpv. Among the groups challenged with K08-1054, only CV/K08-1054 exhibited significantly lower viral loads in the lung compared with the lung viral load observed in Mock/K08-1054. The mean virus titer in the lungs of CV/K08-1054 pigs was even lower than the viral load (10^1.73^ TCID_50_/mL) in the lungs of the K08-1054-homologous group (Figure 9A). The other heterologous groups, VR-2332/K08-1054 and K07-2273/K08-1054, had mean virus titers of 10^2.07^ TCID_50_/mL and 10^1.96^ TCID_50_/mL, respectively, whereas the mock-treated group had the highest viral load in the lungs, 10^2.55^ TCID_50_/mL. The mean virus titers in the lungs of all of the heterologous groups (K08-1054/K07-2273, VR-2332/K07-2273, and CV/K07-2273) challenged with K07-2273 were significantly lower than the mean virus titer in Mock/K07-2273, whereas the K07-2273 homologous group exhibited a relatively higher residual viral lung load of 10^2.04^ TCID_50_/mL (Figure 9B). A very high residual viral load was also observed in the lungs of the mock-treated group, with a titer of 10^4.28^ TCID_50_/mL.

## 4. Discussion

Current MLVs provide effective protection against homologous challenges but lack proper cross-protection against heterologous challenges [28]. Therefore, several reverse genetics-based PRRSV chimeras have been constructed and evaluated for their enhanced cross-protection range in previous studies. A recent study has demonstrated that chimeric PRRS viruses harboring mixed structural genes from two different PRRSV strains can provide protection against both donor viruses [29]. A study has also reported a cross-neutralizing antibody response from a PRRSV chimera engineered to contain Korean strain-specific (LMY) structural genes in an FL12 backbone [30]. However, it has not been further evaluated in pigs. Moreover, the presence of single, strain-specific neutralizing epitopes in a PRRSV might limit its cross-protection against genetically diverse PRRSVs [31]. Another study demonstrated the construction of structural genes-shuffled chimeric PRRSVs, and one chimera and its parental strain were observed to offer partial cross-protection in pigs [32]. Although the random shuffling of structural genes increases the heterogeneity of neutralizing epitopes in the resulting PRRSV construct, the significance of structural proteins from different PRRSV strains and their proper frame/order cannot be neglected when constructing an organized vaccine platform. Recently, a new study demonstrated strong heterologous protection conferred by a synthetic PRRSV construct with a consensus sequence obtained from the whole-genome sequence alignment of 59 different PRRSVs [33]. Although it might be an efficient approach to broaden cross-protection against various PRRSV strains, genome-wide sequence manipulations along with an inability to include or exclude immunemodulatory sequences might render it a less effective platform for vaccine development. Due to these shortcomings, it becomes imperative to construct an organized and simple PRRSV vaccine platform that can be easily manipulated into a region or farm-specific vaccine with a backbone of highly immunogenic PRRSV strains containing overlapping ORFs 3–4 and heterodimer-coding ORFs 5–6 from two different field isolates. Because the higher immune induction of ORFs 1a and 7 from FL12 was demonstrated in our previous study [34], FL12 was used as a backbone to construct a chimeric virus containing ORFs 3–4 and ORFs 5–6 from two Korean strains (K08-1054 and K07-2273, respectively), and evaluated for cross-protective efficacy in pigs.

One of the major challenges to PRRS vaccinology is that various PRRSV strains induce different levels of SVN antibodies, even against homologous virus [17], which was also evident in this study among the PRRVs evaluated for SVN antibody titers at 21 dpv. In this study, CV-vaccinated pigs displayed high SVN antibody titers of 1:14 ± 9.1 and 1:56 ± 36.6 against K08-1054 and K07-2273, respectively; besides exhibiting a significant reduction in viremia compared with the mock groups at 42 dpv. This specific result agrees with a previous report demonstrating that an SVN antibody titer of 1:8 was sufficient to block viremia in PRRS-infected pigs [35]. Moreover, to attain sterilizing immunity against PRRS infection, a vaccine should induce SVN antibodies to a titer of 1:32, as concluded in previous studies [35,36]. The results of this study demonstrated that 50% of pigs vaccinated with CV exhibited an SVN antibody titer of ≥1:32 against the Korean strains.

Cross-neutralization between various PRRSV strains is believed to be dependent on the homologies of the structural proteins (ORFs 2–6) [22,37,38,39,40,41,42,43,44,45]. This observation agrees with this study, in which higher SVN antibody induction was seen at 42 dpv in CV/K08-1054 and CV/K07-2273 against K08-1054 and K07-2273, respectively, compared with the other groups. The reason for the higher SVN antibody induction in these CV groups might be due to the ORFs 3–4 and ORFs 5–6 from K08-1054 and K07-2273, respectively [22,37,38,39,40,41,42,43,44,45]. Notably, a major role for ORFs 5–6 in virus neutralization has been examined in previous studies [10,39,44,46,47,48,49,50], which might explain the high SVN antibody induction in CV/K07-2273 against K07-2273. However, in this study, CV/K07-2273 exhibited nine-fold higher SVN antibody titers against K07-2273 compared with the antibody titers induced in the homologous group (K07-2273/K07-2273), indicating a possible role for the CV backbone in SVN antibody induction. It has been previously demonstrated that JA142, which is highly homologous to FL12, the backbone virus for CV, induced high levels of SVN antibody compared with other PRRSV strains [17]. However, the association between SVN antibody induction and viremia prevention in PRRS infection is still debatable [51], as some studies suggest no association between PRRSV-induced SVN antibody titer and viremia level in PRRS-infected pigs [35,52,53,54]. These observations are consistent with the present study. CV exhibited efficient viremia reduction and high SVN antibody induction after vaccination and challenge, whereas other PRRSVs (K08-1054, K07-2273, and VR-2332) induced lower SVN antibody titers despite fair reduction of viremia.

To date, the cell-mediated immune response elicited by PRRSV is unclear. Previous reports have demonstrated that PRRSV caused immunosuppression by upregulating IL-10, which in turn inhibited the expression of the cytokines responsible for viral clearance (IFN-α, IFN-γ, IL-12, and TNF-α) [54,55]. In this study, CV-vaccinated pigs presented a different scenario compared to other groups, with relatively lower IL-10, higher IFN-γ and higher TNF-α mRNA levels in PBMC cultures at 21 dpv. Simultaneously, higher TNF-α and IL-12 protein expression in PBMC supernatants suggests that CV induced the least immunosuppressive effect among all of the vaccines tested. These results are also consistent with a study in which high and spontaneous IFN-γ production in cultures was observed in unstimulated pig PBMCs inoculated with an inactivated vaccine [56]. Moreover, the higher TNF-α and IFN-γ expression in CV-vaccinated pigs in this study was also associated with reduction of viremia at 21 dpv, which is in agreement with earlier studies that demonstrated roles for TNF-α and IFN-γ in the inhibition of PRRSV replication [57,58].

Chimeric PRRS viruses used in previous animal studies have not been shown to affect the body weight of infected pigs [20,59], which is consistent with this study, in which CV-vaccinated pigs displayed fair weight gain at 25 dpv compared to the other vaccinated groups. However, the CV-vaccinated groups exhibited similar or relatively higher gross lung pathology compared with the other vaccinated groups despite displaying significantly higher SVN antibodies against homologous virus, cross-neutralization titers and viremia reduction that were comparable to those observed in homologous-challenged Korean strains, with significantly lower IL-10 expression in PBMCs at 21 dpv. This observation is in agreement with a recent study that demonstrated an enhanced adaptive immune response and virus clearance coupled with increased gross pathology in pigs that were experimentally infected with an SU-1 bell strain of type-I PRRSV [60].

## 5. Conclusions

In summary, CV-vaccinated pigs exhibited reduced viremia and viral lung loads against challenges with two heterologous viruses, which induced relatively higher levels of SVN antibody and cell-mediated immune responses compared with the other vaccinated groups. The study also suggests that the cross-protective capacity of CV tested against K08-1054 and K07-2273 might be facilitated due not only to the presence of structural proteins from these virus strains but also by an immunogenic pFL-12 backbone. Therefore, this study opens new possibilities for broadly effective vaccines against various PRRSV strains.

## Figures and Tables

**Table 1:** Open reading frame (ORF)-wise percentage of amino acid homology between the different viruses used in this study.

**Divergence**	**Percentage of Homology**																				
		**ORF 3**					**ORF 4**					**ORF 5**					**ORF 6**				
		**a**	**b**	**c**	**d**	**e**	**a**	**b**	**c**	**d**	**e**	**a**	**b**	**c**	**d**	**e**	**a**	**b**	**c**	**d**	**e**
	**a**		94.1	99.6	85.1	88.2		96.6	99.4	66.3	88.3		96.5	85.1	85.1	91.0		98.3	94.9	94.4	96.6
	**b**	5.7		94.1	86.3	87.5	2.8		96.6	65.7	88.8	3.0		84.6	84.6	90.5	1.2		94.9	94.9	97.1
	**c**	0.0	5.7		85.1	88.2	0.0	2.8		66.3	88.3	16.1	16.7		99.5	88.1	4.7	4.7		99.4	94.9
	**d**	16.2	14.7	16.2		83.5	43.6	44.6	43.6		66.9	16.1	16.7	0.0		88.1	4.7	4.7	0.0		94.9
	**e**	12.4	13.3	12.4	18.1		12.1	11.5	12.1	43.6		9.0	9.6	12.4	12.4		2.9	2.3	4.7	4.7	

Different letters signify different viruses used in this study (a: K08-1054; b: VR-2332; c: chimeric virus (CV); d: K07-2273; e: FL-12).

**Table 2:** Primers used in the construction of the chimeric virus.

Primer Name	Primers (5′–3′)	Sequence Origin	Nucleotide Position/Ref.	Purpose
SphI/BsrGI-F	*GCATGC*GTGTCAAAGCT*T**GTACA*****TTCCTCCATATTTTCCTCC**	Virus	[21]	Shuttle vector construction
SpeIpolyA-R	*ACTAGT*GTGTCAGTCGACGCG**TTTTTTTTTTTTTTTTTTTTTTTTTTTTTTTTTTTTTTTTTAATTTCGGCCGCATGGTTCTCGC**	Virus	[21]	Shuttle vector construction
N_K08-1054_ORF3F	**TCCTCCATATTCTCCTCTGTT**	Virus	12,717–12,737	Gene Swap
N_K08-1054_ORF3R	**TGGTGTTGGTCTCAATGTCTGC**	Virus	13,336–13,357	Gene Swap
N_K08-1054_ORF4F	**TTGGTTTCTCAGGCGTTC**	Virus	13,288–13,305	Gene Swap
N_K08-1054_ORF4R	**CCCCAACATACTTAAACA**	Virus	13,781–13,798	Gene Swap
N_K07-2273_ORF5F	**ATGTTGGGGAAATGCTTGAC**	Virus	13,790–13,809	Gene Swap
N_K07-2273_ORF5R	**GAAAACGCCAAAACTACCT**	Virus	14,426–14,444	Gene Swap
N_K07-2273_ORF6F	**GTCCCTAGACGACTTTTG**	Virus	14,385–14,402	Gene Swap
N_K07-2273_ORF6R	**CTTGCCGTTGTTATTTGG**	Virus	14,894–14,911	Gene Swap
PmeI-F	GGTTTTCCCAGTCACGACGT*GTTTAAAC*GACGGCCAGTGAATTG	Vector	2944–2987	Site-Directed Mutagenesis
PmeI-R	CAATTCACTGGCCGTC*GTTTAAAC*ACGTCGTGACTGGGAAAACC	Vector	2944–2987	Site-Directed Mutagenesis
PacI-F	CTAATGAGTGAGCTAACTCAGA*TTAATTAA*GATTGGGCTCACTGCC	Vector	252–296	Site-Directed Mutagenesis
PacI-R	GGCAGTGAGCCCAATC*TTAATTAA*TCTGAGTTAGCTCACTCATTAG	Vector	252–296	Site-Directed Mutagenesis
AflII-PacI F	**CCAAATAACAACGGCAAG**	Virus	15,665–15,682	Shuttle vector construction
AflII-PacI R	ATGAACAAACGACCCAACA	Vector ^†^	1319–1337	Shuttle vector construction
HpaI-BsrGI F	**GATGGTCTGGAAGGACAAG**	Virus	11,572–11,590	Site-Directed Mutagenesis
HpaI-BsrGI R	**ATGGAGGAG*TGTACA*GCTATTAG**	Virus	12,702–12,724	Site-Directed Mutagenesis

Nucleotide sequences in bold letters represent PRRS virus-specific sequences. Nucleotide sequences in italics represent restriction enzyme recognition sites; ^†^ pOptiVEC™-TOPO^®^ TA cloning kit vector, pGEM^®^-T Easy vector. F: forward; R: reverse.

**Table 3:** Primers used to measure the mRNA expression levels of various cytokines.

Genes	Forward Primer (5′–3′)	Reverse Primer (5′–3′)	* Accession/Ref.
β-Actin	GCGGGACATCAAGGAGAAG	AGGAAGGAGGGCTGGAAGAG	U07786
TNF-α	TTATTCAGGAGGGCGAGGT	AGCAAAAGGAGGCACAGAGG	NM214022
IFN-α	TCTCATGCACCAGAGCCA	CCTGGACCACAGAAGGGA	[26]
IL-10	TGACGATGAAGATGAGGAAGAA	GAACCTTGGAGCAGATTTTGA	NM_214041
IL-12	TCAGGGACATCATCAAACCA	GAACACCAAACATCAGGGAAA	NM214013
IFN-γ	GACTTTGTGTTTTTCTGGCTCTTAC	TTTTGTCACTCTCCTCTTTCCA	NM_213948

TNF: tumor necrosis factor; IFN: interferon; IL: interleukin; * GenBank Accession number.
